# Rs7193343 polymorphism in zinc finger homeobox 3 (ZFHX3) gene and atrial fibrillation: an updated meta-analysis of 10 case-control comparisons

**DOI:** 10.1186/s12872-015-0044-y

**Published:** 2015-06-26

**Authors:** ChuanNan Zhai, HongLiang Cong, YuJie Liu, Ying Zhang, XianFeng Liu, Hao Zhang, ZhiJing Ren

**Affiliations:** Department of Cardiology, Tianjin Chest Hospital, Taierzhuang South Road No. 291, Jinnan District Tianjin, 300350, China; Graduate School, Tianjin Medical University, Tianjin, 300051, China; Department of Cardiology, Tianjin Gongan Hospital, Xinhua Road No. 162, Heping District Tianjin, 300042, China

**Keywords:** Atrial fibrillation, Meta-analysis, Zinc finger homeobox 3 gene, Polymorphism, Rs7193343

## Abstract

**Background:**

The previous genome-wide studies have shown that rs7193343 single-nucleotide polymorphism (SNP) in zinc finger homeobox 3 (ZFHX3) gene correlate with risk of atrial fibrillation (AF). However, the distribution of this SNP differs significantly among various populations. The present study was to investigate whether combined evidence shows the association between ZFHX3 rs7193343 SNP and the risk of AF in various populations.

**Methods:**

A systematic search of all studies published through Dec 2014 was conducted using the Medline, Embase, WanFang, ScienceDirect, CNKI, and OVID databases. The case-control studies that evaluated an association between rs7193343 SNP and risk of AF were identified. The association between the ZFHX3 rs7193343 SNP and AF susceptibility was assessed using genetic models.

**Results:**

We collected 10 comparisons from six studies for rs7193343 SNP, including 1037 cases and 4310 controls in Asian, 5583 cases and 38215 controls in Caucasian, and then performed an updated meta-analysis and subgroup analysis based on ethnicity. In overall population, the occurrence of AF was found to be associated with T-allelic of rs7193343 SNP in ZFHX3 (OR =1.17, 95% CI 1.10-1.26). In subgroup analysis, we observed there was significant association between T-allele of rs7193343 and risk of AF in Caucasian subgroups (OR =1.20, 95% CI 1.12-1.30), but no statistically significance (OR = 1.07, 95% CI 0.92-1.24) in Asian population.

**Conclusion:**

In Caucasian population, genetic variant rs7193343 SNP is associated with risk of AF in Caucasian population. However, no association is found in Asian population based on the current evidence. Further studies with larger sample size involving case-control populations with multiple ethnics are still required in the future.

## Background

Atrial fibrillation (AF) is the most common sustained cardiac arrhythmia in clinical practice, and affect individuals suffer from increased rates of stroke, and lead to higher risk of incidence and mortality of cardiovascular disease [1]. The incidence of AF has estimated rate of 0.4–1.0 % which increases with age, in the general population [2, 3]. In 80 people over the age, its prevalence is high at 7.5 % [4, 5]. The factors that increase the risk of developing AF include age, hypertension, heart failure, structural heart diseases, valvular heart disease and a variety of other factors [3, 6]. However, contemporary clinical treatment strategies have only confined curative effects that likely stem from our limited understanding of its potential pathophysiology. Therefore, most medical experts tried to find the significant elements with risk of AF.

Recently, studies have provided unequivocal evidence that AF has the important relevance of genetic factors [7]. AF has been found to occur in large families (monogenic AF) and can be inherited in either an autosomal dominant model or an autosomal recessive [8]. Several genetic loci, such as loci on chromosome 1q21, 3q21, 5p13, 11p15.5, 12p13, 21q22 and 17q23–q24, have been identified for monogenetic AF [9]. Previous studies shown that the 3 loci most strongly associated with AF occur on chromosomes 4q25 (near PITX2) [10], 16q22 (in ZFHX3) [11], and 1q21 (in KCNN3) [12]. A recent genome-wide association study (GWAS) has also identified variants on chromosome 16q22. The proximate study investigated the role of genetic variants of ZFHX3 (zinc finger homeobox 3) in AF, single-nucleotide polymorphism (SNPs) rs2106261 and rs6499600 showed significant associations, and rs16971436 conferred a borderline significant association with risk of AF in Chinese Han populations [13]. SNP rs7193343 in ZFHX3 gene has been pointed out as marker strongly associated with AF in several different populations [14–16], while other studies assessed the association of rs7193343 with susceptibility of AF, which shown that the association was not statistically significant [14, 16]. Therefore, the results are still in controversy.

Therefore, the aim of the present meta-analysis was to investigate whether combined evidence shows the association between ZFHX3 rs7193343 polymorphism and the risk of AF in various populations, determining whether there was heterogeneity among the studies.

## Methods

### Search strategy

We performed a systematic search of Medline, Embase, WanFang, ScienceDirect, CNKI, and OVID to identify published epidemiological studies through Dec 2014 that were related to the rs7193343 ZFHX3 polymorphism and AF. The medical subject headings (MeSH; National Library of Medicine, Bethesda, Maryland) “zinc finger homeobox 3”, “genetic polymorphism”, “atrial fibrillation”, and the free-text words “ZFHX3” or “rs7193343” were combined. Only studies published in English or in Chinese were included in the present study. Furthermore, the reference lists of all of the full text papers were examined to identify any initially omitted studies. Secondary searches of the grey literature were not performed.

### Inclusion and exclusion criteria

Articles from peer-reviewed medical journals were included if they reported on studies using case-control, nested case-control, cross-sectional design, or cohort and provided sufficient data to calculate an odds ratio (OR) and corresponding 95 % confidence interval (CI). After that, comparisons of laboratory methods and overlapping study data were excluded. Using Hardy-Weinberg equilibrium (HWE), we excluded those studies that contain genotype frequencies that weren’t meet criteria in the control groups.

### Study selection

Two reviewers independently screened the titles and abstracts for the eligibility criteria. Subsequently, reviewers read the full text of the studies that potentially met the inclusion criteria, and the literature was reviewed to determine final inclusive data. If inclusions have disagreements, we reached a consensus through discussion.

### Date extraction

With a standard data extraction form used, two of the authors independently extracted the following data from each full-text report. The data extracted from the candidate studies included the title, authors, published year, number of cases or controls, ethnicity, age, gender, study design, genotyping method, genotype distribution, and frequency of T-allele of the rs7193343 polymorphism in cases or controls. We also examined whether the genotype distributions of the control groups followed HWE [17].

### Statistical analysis

Data analysis was conducted using STATA 12.0 (Statacorp, college station, Tex). The association between the ZFHX3 rs7193343 polymorphism and AF susceptibility was assessed under the following genetic models, which were treated as a dichotomous variable, T-allele versus C-allele for allele level comparison.

Between-study heterogeneity was tested using Q statistics, and P < 0.1 was considered statistically significant. The Mantel-Haenszel method for fixed effects and the Der-Simonian and Laird method for random effects were used to estimate pooled effects [18]. We used fixed-effects methods if the result of the Q test was not significant. Otherwise, we calculated the pooled ORs and 95% CIs assuming a random-effects model. Fixed effects assume that genetic factors have similar effects on autoimmune disease susceptibility across all studies and that the observed variations between studies are caused by chance alone [19]. The random effects model assumes that different studies may have substantial diversity and assesses both within- and between-study variation [20]. A recently developed measure, I^2^, was used to quantify the inconsistency among the studies’ results with values of 50 % or higher and the large heterogeneity for values of 75 % or higher [21]. The data are shown as the ORs with 95 %CIs, with two-tailed P-values; statistical significance was set at P < 0.05 (two-tailed).

Publication bias was conducted both visually by using a funnel plot and statistically via Begg funnel plots and Egger’s bias test, which measures the degree of funnel plot asymmetry [22, 23]. The Begg adjusted rank correlation test was used to assess the correlation between test accuracy estimates and their variances. The deviation of Spearman’s rho values from zero provides an estimate of funnel plot asymmetry. Positive values indicate a trend toward higher levels of test accuracy in studies with smaller sample sizes. The Egger’s bias test detects funnel plot asymmetry by determining whether the intercept deviates significantly from zero in a regression of the standardized effect estimates against their precision.

## Results

### Search results

**Fig. 1:**
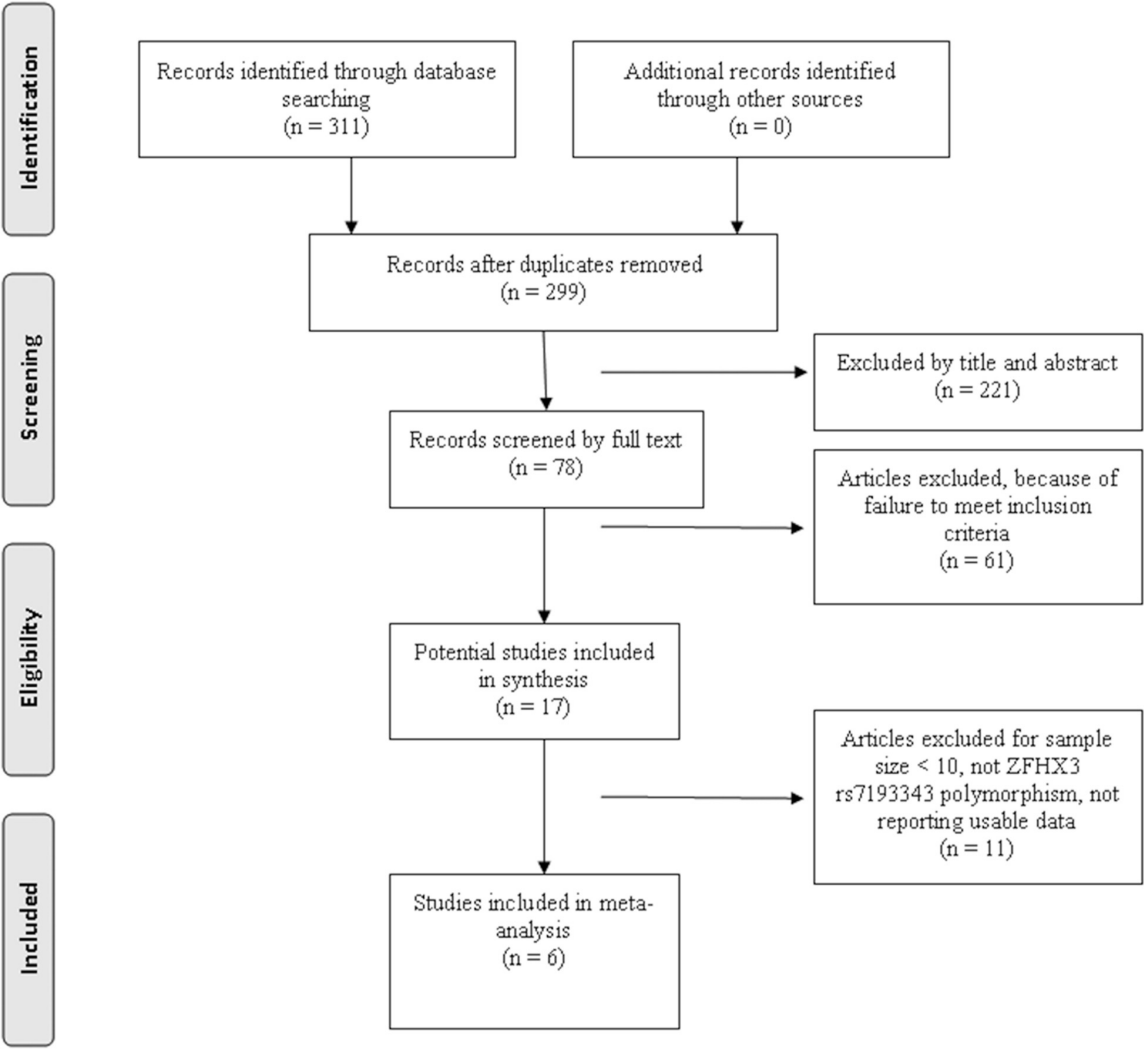
Flow of studies through the meta-analysis

We initially obtained 311 potential articles, and after screened abstract, among which most were excluded for no relevance to our analysis. Eleven articles then were removed because small number cases and unusable data. Finally, five studies [14–16, 24, 25] and a Chinese study [26] including 10 comparisons for rs7193343 that all adopted observational study design eventually satisfied the eligibility criteria (Fig. 1).

### Characteristics of included studies

A total of 10 comparisons from six studies for rs7193343 polymorphism were involved in presence updated meta-analysis containing 6620 cases and 42525 controls (including 1037 cases and 4310 controls in Asian, 5583 cases and 38215 controls in Caucasian) (Table 1). One articles as a brief communication was a multi-centers case-control study including 4 comparisons [14]. However we could not obtain genotype information from the above study. The china study [16] also did not provide genotype data. Although we tried to contract authors of the original studies, no response got. The characteristics of included studies were shown in Table 2.

**Table 1:** Allele counts for the rs7193343 polymorphism in the included studies

Study	country	Case					Control				
		Genotype ^a^			Allele ^b^ (%)		Genotype ^a^			Allele ^b^ (%)	
		TT	TC	CC	T	C	TT	TC	CC	T	C
DanielF,2009	Iceland	NA	NA	NA	0.229	0.771	NA	NA	NA	0.199	0.801
DanielF,2009	Iceland	NA	NA	NA	0.238	0.762	NA	NA	NA	0.205	0.795
DanielF,2009	Norway	NA	NA	NA	0.177	0.823	NA	NA	NA	0.166	0.834
DanielF,2009	USA	NA	NA	NA	0.183	0.817	NA	NA	NA	0.139	0.861
DanielF,2009	HongKong	NA	NA	NA	0.686	0.314	NA	NA	NA	0.676	0.324
CongL,2011	China	NA	NA	NA	0.320	0.680	NA	NA	NA	0.320	0.680
Marek,2011	Poland	27	128	230	0.222	0.778	28	148	344	0.185	0.815
Wang,2012	China	22	68	12	0.549	0.451	10	66	24	0.430	0.570
Parvez,2013	USA	5	40	57	0.231	0.769	9	62	97	0.217	0.783
Albert,2014	Spanish	10	88	159	0.210	0.790	10	106	263	0.166	0.834

*NA* not available

^a^ Number of [homozygotes of risk allele, TT]/[heterozygotes, TC]/[homozygotes of the other allele, CC]

^b^ Risk allele (T-allele or C- allele) frequency

**Table 2:** Characteristics of included studies

Study,year	ethnicity	Characteristics of controls	Genotyp-ing methods	No.case of total AF	No.case of lone AF	No. of control	age		Gender(M/F)		H W E
							Case	Control	Case	Control	
DanielF,2009	Iceland	Health	HumanHap	2381	NA	33723	NA		NA		Y
DanielF,2009	Iceland	Health	HumanHap	970	NA	1939	NA		NA		Y
DanielF,2009	Norway	Health	HumanHap	722	NA	711	NA		NA		Y
DanielF,2009	USA	Health	HumanHap	735	NA	729	NA		NA		Y
DanielF,2009	HongKong	Health	HumanHap	285	NA	2763	NA		NA		Y
CongL,2011	China	Health	PCR	650	180	1447	58.4(15.9)	59.7(12.2)	398/252	902/545	Y
Marek,2011	Poland	AF^a^	Taqman	410^b^	NA	550^b^	53.3(11.3)	53.1(10.4)	272/128	331/169	Y
Wang,2012	China	Health	PCR	102	NA	100	61.5(12.5)	60.4(9.9)	56/46	52/48	Y
Parvez,2013	USA	AF^a^	PCR	108^b^	NA	184^b^	67(59-72)	66(58-72)	82/26	142/42	Y
Albert,2014	Spanish	Health	Taqman	257	123	379	60.6(11.5)	42.6(15.2)	148/109	193/186	Y

*NA* not available

AF^a^ mean AF recurrence after ablation

^b^mean about the numbers of AF recurrence after ablation

### Association of rs7193343 polymorphism of ZFHX3 gene with the risk of AF in overall population

**Fig. 2:**
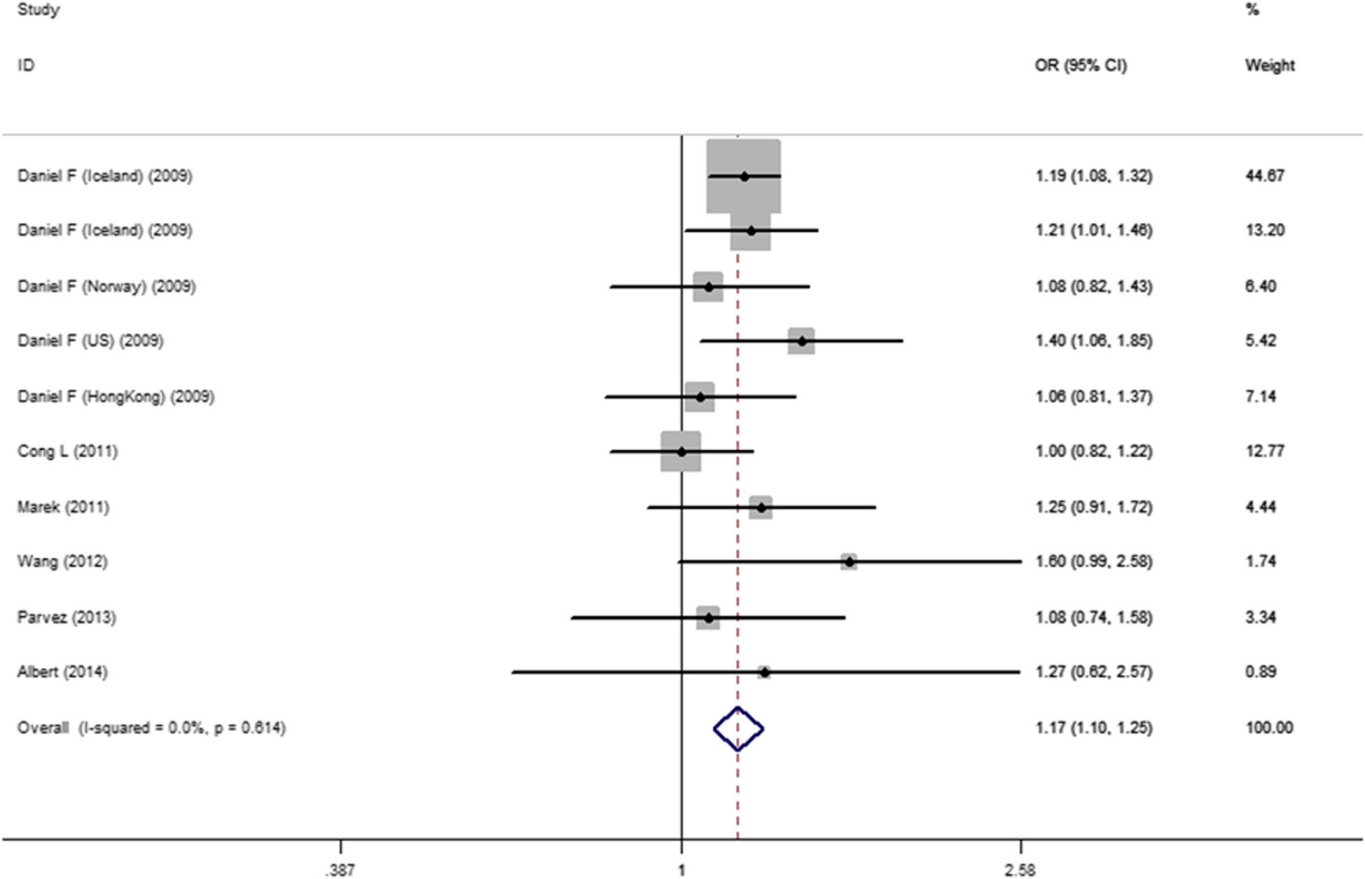
ORs and 95 %CI of individual studies and pooled data for overall study of the association between the ZFHX3 rs7193343 T-allele and AF

As shown in Fig. 2, we used a fixed-effects model to analysis overall studies data of 10 case-control comparisons, performed for meta-analysis and generated a combined allelic OR 1.17 of for risk allele (95 % CI 1.10-1.26), identified no statistics heterogeneity (Q = 7.22, I^2^ = 0.0 %). These results indicated that there was statistically significant in the association between rs7193343 and AF.

### Association of rs7193343 polymorphism of ZFHX3 gene with the risk of AF in subgroups analysis

**Fig. 3:**
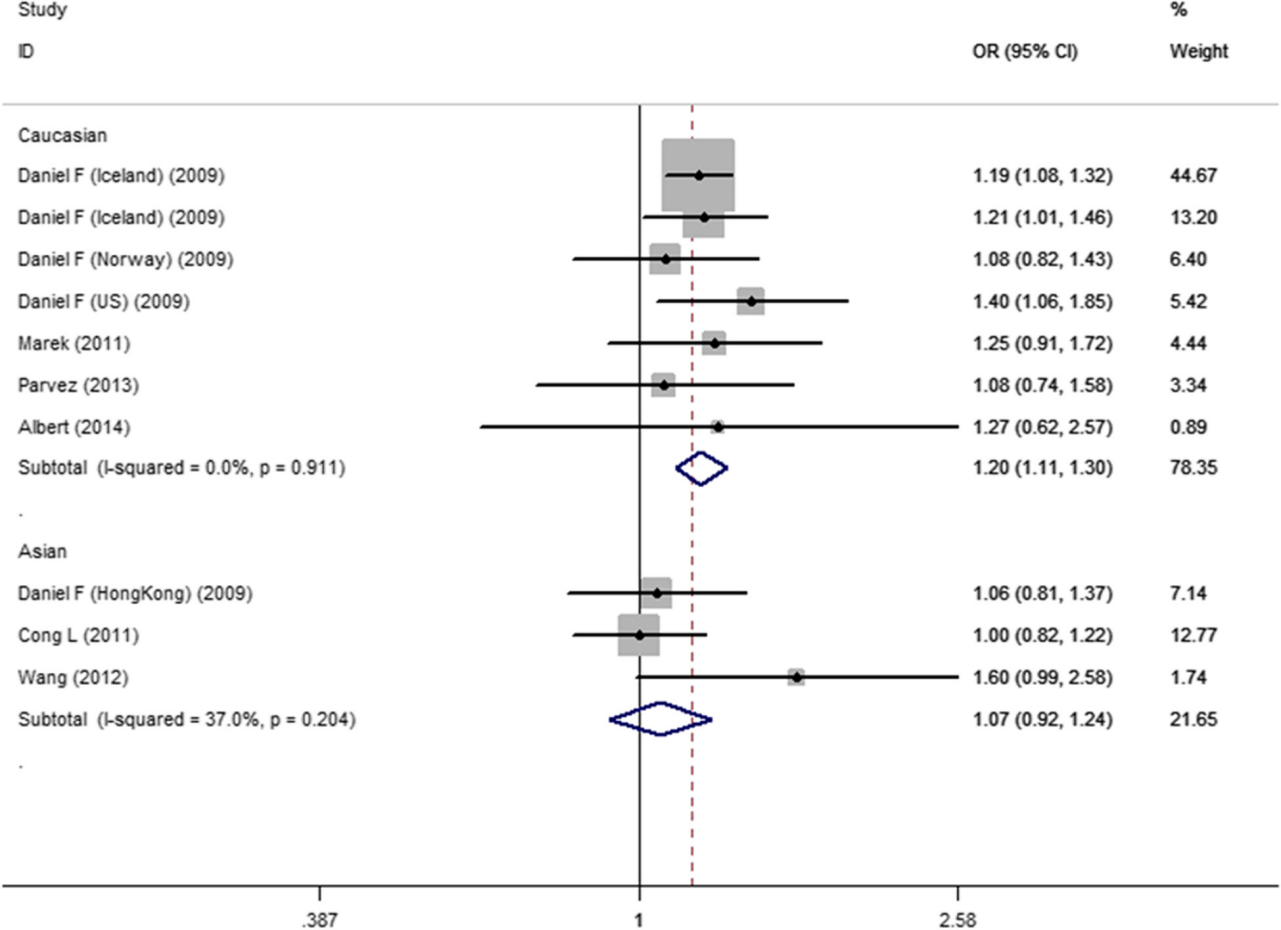
ORs and 95 %CI of individual studies and pooled data for stratification study of the association between the ZFHX3 rs7193343 T-allele and AF

As shown in Fig. 3, when we restricted to ethnicity subgroup analysis, there was significant association between rs7193343 and the risk of AF in Caucasian subgroups (OR =1.20, 95 %CI 1.12-1.30) in the fixed-effects model, and the result shown no obvious statistics heterogeneity (Q = 2.09, I^2^ = 0.0 %). Three studies reported the association between rs7193343 polymorphism and AF in Asian population, we used a fixed-effects model analysis because there was no heterogeneity among three studies (Q = 3.18, I^2^ = 37.0 %), shown no statistically significance (OR = 1.07, 95 %CI 0.92-1.24) (Table 3).

**Table 3:** Summary ORs and 95% CIs of the rs7193343 polymorphism in ZFHX3 and AF susceptibility

Comparison	Population	Sample size		No. of studies	Type of model	Test of association			Test of heterogeneity		
		Case	Control			OR	95% CI	P value	Q test	P value	I^2^
T allele versus C allele	Overall	6620	42525	10	Fixed	1.17	1.10-1.26	<0.01	7.22	0.61	0.0 %
	Asian	1037	4310	3	Fixed	1.07	0.92-1.24	0.40	3.18	0.20	37.0 %
	Caucasian	5583	38215	7	Fixed	1.20	1.12-1.30	<0.01	2.09	0.91	0.0 %

### Publication bias

**Fig. 4:**
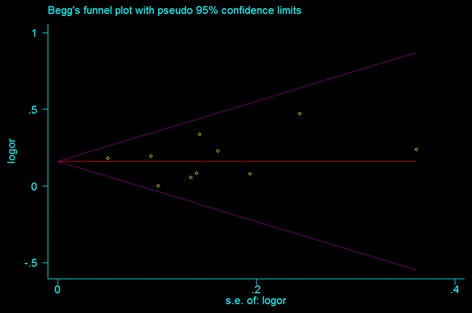
Funnel plot of studies for the association between the ZFHX3 rs7193343 T-allele and AF in all subjects (Begg rank correlation test, P = 0.47)

**Fig. 5:**
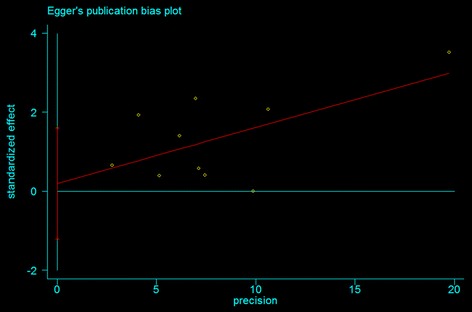
Funnel plot of studies for the association between the ZFHX3 rs7193343 T-allele and AF in all subjects (Egger weighted regression test, P = 0.76)

The publication bias test was performed for overall populations. Significant publication bias was shown for overall populations by the Begg rank correlation method (P = 0.47) (Fig. 4) and the Egger weighted regression method (P = 0.76) (Fig. 5).

## Discussion

Atrial fibrillation (AF) represents the most serious sustained arrhythmia in the process of clinical diagnosis and treatments. It is the feature of uncoordinated atrial activation. Some studies shown that AF was a important factor with a lifetime risk of one in four for men and women 40 years of age and older [27]. Currently, a number of studies showed that AF was in the relationship of SNPs in genes [13, 28, 29]. A SNP, rs7193343, had been investigated widely. Although several studies demonstrated that rs7193343 polymorphism was associated with occurrence of AF, the results were still in controversy. We, therefore, conducted the present meta-analysis to evaluate the relationship of rs7193343 polymorphism and susceptibility of AF. Thus, we aim to provide objective evidence in the investigation of rs7193343 polymorphism in patients with AF, based on the current published studies.

Recently, a growing body of researches provided the relationship of a variant in the ZFHX3 gene on chromosome 16q22, rs7193343-T and AF in multiple population of different ancestry. However, their conclusions were not consistent. The distribution of genotype of rs7193343 polymorphism in ZFHX3 gene differed significantly among populations. Gudbjartsson et al. [14] expanded a genome-wide association study (GWAS) on atrial fibrillation, they previously identified risk variant on 16q22 rs7193343 associated significantly with AF in samples from Iceland, Norway and US, they also illustrated that the association of SNPs rs7193343-T with AF in Chinese population from Hong Kong, but the result was not significant in this cohort. Furthermore, they found that the T allele of rs7193343 is obviously much more frequent in Chinese descent than the samples of European population. Soon afterwards, the study of Cong L et. al [16] provided the first evidence of a cross-race susceptibility of the 16q22 AF locus in a Chinese Han population, and expanded the association between ZFHX3 and AF to a non-European ancestry population. They carried out a large-scale case-control association study, and identified that ZFHX3 SNP rs7193343 was not associated with AF in the Chinese Han population. Kiliszek et al. [15] identified SNP rs7193343 polymorphism correlated significantly with AF in Polish patients, and confirmed rs7193343 on chromosome 16q22 that it was an independent marker of AF. Although another study evaluated genomic markers which can predict timing of AF recurrence in patients undergoing elective direct current cardioversion, it did not confirm that rs7193343 has association with early AF recurrence [24].

The present study demonstrated that there was statistically significant in the association between T-allele frequency of rs7193343 and AF in overall population. Interestingly, subgroup analysis was performed according to the ethnicity, showing significant association between T-allele frequency of rs7193343 and AF in Caucasian population but not in Asian population. Although a previous meta-analysis was conducted recently with positive results [25], no subgroup analysis was performed based on the ethnicity. The heterogeneity originated from ethnicity was of great importance in the pooled results. Thus, it was imperative to update the previous meta-analysis in accordance with ethnicity of individual study. The pooled result based on one kind of ethnicity may be more stable. Although the pooled results in overall population of the present study was consistent with the previous one, there was no statistically significant difference in Asian population. The subgroup analysis is the strengthen of the present meta-analysis.

Rs7193343 is an intronic SNP located in the zinc finger homeobox 3 (ZFHX3) gene (chromosome 16q22) [14]. The variant is also called AT motif-binding factor 1 (ATBF1). The same variant was associated with Kawasaki disease, an inflammatory vasculitis predominantly seen in young children. The gene encodes a transcription factor named Atbf1 that was first described as an enhancer of human a-fetoprotein (AFP) gene expression in the liver [30]. This gene regulates neuronal and muscle differentiation and is a tumor suppressor gene in several types of cancer [31]. ZFHX3 is expressed in various tissues, including heart, liver, lung, kidney, pituitary gland and brain. Although it is expressed in mouse heart [32], its function in heart tissue is unknown. Atbf1 is required for early transcriptional activation of the gene POU class 1 homeobox 1 (POU1F1) that regulates pituitary cell differentiation and hormone expression in mammals [33]. POU1F1 interacts with the paired-like homeodomain transcription factor 2 (PITX2) to facilitate DNA binding and transcriptional activity, which is of interest because the previously identified AF variants on chromosome 4q25 are located close to PITX2, a gene critical for heart development [34]. Therefore, we speculate there is an association between rs7193343 and expression of ZFHX3 in heart tissue. The above mentioned may be the mechanism of the association between ZFHX3 rs7193343 polymorphism and the risk of AF.

The following potential factors may be account for the different pooled results between the above two ethnical group: (1) inconsistence in genetic background [35], (2) different habits in individual ethnicity [36], (3) environmental factors leading to different susceptibility of AF [37]. Furthermore, we also speculate that the reason why the statistical difference was not significant in Asian population after subgroup analysis may be the limited sample size in this population. Therefore, more studies with large sample size are required to conduct further analysis, especially in Asian population.

Although the results of test for heterogeneity were not significant for subgroup population, heterogeneity cannot be completely resolved. The heterogeneity of included studies includes the following: quality of included studies, gender proportion, age, classification of AF, different genetic background, environmental factors, sampling criteria and cultural difference. The heterogeneity of genetic effects between individual studies may also be caused by the existence of gene-environmental or genetic interaction. In spite of only ten comparisons included, the pooled results may have clinical significance according to the current evidence. Although no publication bias was found in the present study, it is also important to bear in mind that publication bias may exist, since the negative results are less likely to be published. Accordingly, while the results of this meta-analysis should be considered appropriate, the above methodological defects should be considered when interpreting the findings. The present study could provide objective evidence by using genetic meta-analysis approach.

The primary limitations of this updated meta-analysis include the following: (1) the statistical efficacy could be improved by including more studies; (2) Some non-English publications not being included in this study may have caused important studies to be overlooked and publication bias from significant conclusions being more easily published; (3) The genotyping data of SNPs rs7193343 were insufficient, so we were unable to conduct meta-analysis based on the limited studies data; (4) the combined analysis of the results may be affected by the impact of clinical heterogeneity, including the general condition of the cases, habits, medical history, medication compliance and other factors.

## Conclusion

Genetic variant rs7193343 is associated with a crucial risk of AF in Caucasian population, whereas SNP rs7193343 is not association with AF in Asian population. This variant could have a clinical utility in estimating AF risk. On the other hand, more studies with great sample size and multiple ethnics are required to determine the significance of rs7193343 SNP between them and to define the effect of the size of T-allele on susceptibility to AF.

### Ethical approval

Ethical approval was not required for this study design.

## References

[1] Wolf PA, Abbott RD, Kannel WB (1991). Atrial fibrillation as an independent risk factor for stroke: the Framingham Study. Stroke.

[2] Feinberg WM, Blackshear JL, Laupacis A, Kronmal R, Hart RG (1995). Prevalence, age distribution, and gender of patients with atrial fibrillation. Analysis and implications. Arch Intern Med.

[3] Fuster V, Ryden LE, Cannom DS, Crijns HJ, Curtis AB, Ellenbogen KA, Halperin JL, Le Heuzey JY, Kay GN, Lowe JE (2006). ACC/AHA/ESC 2006 guidelines for the management of patients with atrial fibrillation: full text: a report of the American College of Cardiology/American Heart Association Task Force on practice guidelines and the European Society of Cardiology Committee for Practice Guidelines (Writing Committee to Revise the 2001 guidelines for the management of patients with atrial fibrillation) developed in collaboration with the European Heart Rhythm Association and the Heart Rhythm Society. Europace.

[4] Stewart S, Hart CL, Hole DJ, McMurray JJ (2001). Population prevalence, incidence, and predictors of atrial fibrillation in the Renfrew/Paisley study. Heart.

[5] Go AS, Hylek EM, Phillips KA, Chang Y, Henault LE, Selby JV, Singer DE (2001). Prevalence of diagnosed atrial fibrillation in adults: national implications for rhythm management and stroke prevention: the AnTicoagulation and Risk Factors in Atrial Fibrillation (ATRIA) Study. JAMA.

[6] Perticone F, Sciacqua A, Perticone M, Tassone EJ, Sesti G, Violi F, Lip GY (2014). Clinical risk factors and subclinical target organ damage as predictors of new-onset of atrial fibrillation: The Catanzaro atrial fibrillation project. Int J Cardiol.

[7] Andalib A, Brugada R, Nattel S (2008). Atrial fibrillation: evidence for genetically determined disease. Curr Opin Cardiol.

[8] Oberti C, Wang L, Li L, Dong J, Rao S, Du W, Wang Q (2004). Genome-wide linkage scan identifies a novel genetic locus on chromosome 5p13 for neonatal atrial fibrillation associated with sudden death and variable cardiomyopathy. Circulation.

[9] Tsai CT, Lai LP, Hwang JJ, Lin JL, Chiang FT (2008). Molecular genetics of atrial fibrillation. J Am Coll Cardiol.

[10] Gudbjartsson DF, Arnar DO, Helgadottir A, Gretarsdottir S, Holm H, Sigurdsson A, Jonasdottir A, Baker A, Thorleifsson G, Kristjansson K (2007). Variants conferring risk of atrial fibrillation on chromosome 4q25. Nature.

[11] Benjamin EJ, Rice KM, Arking DE, Pfeufer A, van Noord C, Smith AV, Schnabel RB, Bis JC, Boerwinkle E, Sinner MF (2009). Variants in ZFHX3 are associated with atrial fibrillation in individuals of European ancestry. Nat Genet.

[12] Ellinor PT, Lunetta KL, Glazer NL, Pfeufer A, Alonso A, Chung MK, Sinner MF, de Bakker PI, Mueller M, Lubitz SA (2010). Common variants in KCNN3 are associated with lone atrial fibrillation. Nat Genet.

[13] Liu Y, Ni B, Lin Y, Chen XG, Fang Z, Zhao L, Hu Z, Zhang F (2014). Genetic polymorphisms in ZFHX3 are associated with atrial fibrillation in a Chinese Han population. PLoS One.

[14] Gudbjartsson DF, Holm H, Gretarsdottir S, Thorleifsson G, Walters GB, Thorgeirsson G, Gulcher J, Mathiesen EB, Njolstad I, Nyrnes A (2009). A sequence variant in ZFHX3 on 16q22 associates with atrial fibrillation and ischemic stroke. Nat Genet.

[15] Kiliszek M, Franaszczyk M, Kozluk E, Lodzinski P, Piatkowska A, Broda G, Ploski R, Opolski G (2011). Association between variants on chromosome 4q25, 16q22 and 1q21 and atrial fibrillation in the Polish population. PLoS One.

[16] Li C, Wang F, Yang Y, Fu F, Xu C, Shi L, Li S, Xia Y, Wu G, Cheng X (2011). Significant association of SNP rs2106261 in the ZFHX3 gene with atrial fibrillation in a Chinese Han GeneID population. Hum Genet.

[17] Hernandez JL, Weir BS (1989). A disequilibrium coefficient approach to Hardy-Weinberg testing. Biometrics.

[18] Robins J, Greenland S, Breslow NE (1986). A general estimator for the variance of the Mantel-Haenszel odds ratio. Am J Epidemiol.

[19] Egger M, Smith GD, Phillips AN (1997). Meta-analysis: principles and procedures. BMJ.

[20] DerSimonian R, Laird N (1986). Meta-analysis in clinical trials. Control Clin Trials.

[21] Higgins JP, Thompson SG (2002). Quantifying heterogeneity in a meta-analysis. Stat Med.

[22] Egger M, Davey SG, Schneider M, Minder C (1997). Bias in meta-analysis detected by a simple, graphical test. BMJ.

[23] Begg CB, Mazumdar M (1994). Operating characteristics of a rank correlation test for publication bias. Biometrics.

[24] Parvez B, Shoemaker MB, Muhammad R, Richardson R, Jiang L, Blair MA, Roden DM, Darbar D (2013). Common genetic polymorphism at 4q25 locus predicts atrial fibrillation recurrence after successful cardioversion. Heart Rhythm.

[25] Ferran A, Alegret JM, Subirana I, Aragones G, Lluis-Ganella C, Romero-Menor C, Planas F, Joven J, Elosua R (2014). Association between rs2200733 and rs7193343 genetic variants and atrial fibrillation in a Spanish population, and meta-analysis of previous studies. Rev Esp Cardiol (Engl Ed).

[26] Wang Y, Li Y, Fan J, Xu Y, Chen W, Xiao P, Ling Z, Yin Y (2012). Relationship between rs7193343 polymorphism in the zinc finger homeobox 3 gene and atrial fibrillation. Journal of Clinical Cardiology (China).

[27] Lloyd-Jones DM, Wang TJ, Leip EP, Larson MG, Levy D, Vasan RS, D’Agostino RB, Massaro JM, Beiser A, Wolf PA (2004). Lifetime risk for development of atrial fibrillation: the Framingham Heart Study. Circulation.

[28] Roldan V, Arroyo AB, Salloum-Asfar S, Manzano-Fernandez S, Garcia-Barbera N, Marin F, Vicente V, Gonzalez-Conejero R, Martinez C (2014). Prognostic role of MIR146A polymorphisms for cardiovascular events in atrial fibrillation. Thromb Haemost.

[29] Orenes-Pinero E, Hernandez-Romero D, Romero-Aniorte AI, Martinez M, Garcia-Honrubia A, Caballero L, Garrigos-Gomez N, Andreu-Cayuelas JM, Gonzalez J, Feliu E (2014). Prognostic value of two polymorphisms in non-sarcomeric genes for the development of atrial fibrillation in patients with hypertrophic cardiomyopathy. QJM.

[30] Morinaga T, Yasuda H, Hashimoto T, Higashio K, Tamaoki T (1991). A human alpha-fetoprotein enhancer-binding protein, ATBF1, contains four homeodomains and seventeen zinc fingers. Mol Cell Biol.

[31] Berry FB, Miura Y, Mihara K, Kaspar P, Sakata N, Hashimoto-Tamaoki T, Tamaoki T (2001). Positive and negative regulation of myogenic differentiation of C2C12 cells by isoforms of the multiple homeodomain zinc finger transcription factor ATBF1. J Biol Chem.

[32] Ido A, Miura Y, Watanabe M, Sakai M, Inoue Y, Miki T, Hashimoto T, Morinaga T, Nishi S, Tamaoki T (1996). Cloning of the cDNA encoding the mouse ATBF1 transcription factor. Gene.

[33] Qi Y, Ranish JA, Zhu X, Krones A, Zhang J, Aebersold R, Rose DW, Rosenfeld MG, Carriere C (2008). Atbf1 is required for the Pit1 gene early activation. Proc Natl Acad Sci U S A.

[34] Amendt BA, Sutherland LB, Semina EV, Russo AF (1998). The molecular basis of Rieger syndrome. Analysis of Pitx2 homeodomain protein activities. J Biol Chem.

[35] Andreasen L, Nielsen JB, Olesen MS: Genetic Aspects of Lone Atrial Fibrillation: What Do We Know? Curr Pharm Des. 2015;21(5):667-78 10.2174/1381612820666140825143610 25175087

[36] Suzuki S, Sagara K, Otsuka T, Kano H, Matsuno S, Takai H, Uejima T, Oikawa Y, Koike A, Nagashima K (2013). Effects of smoking habit on the prevalence of atrial fibrillation in Japanese patients with special reference to sex differences. Circ J.

[37] Etzion Y (2012). A stimulating environment for the atrial kick: spinal cord stimulation can inhibit atrial fibrillation. Heart Rhythm.

